# Rapid Nanofabrication of Nanostructured Interdigitated Electrodes (nIDEs) for Long-Term In Vitro Analysis of Human Induced Pluripotent Stem Cell Differentiated Cardiomyocytes

**DOI:** 10.3390/bios8040088

**Published:** 2018-10-11

**Authors:** Cacie Hart, Avra Kundu, Kowsik Kumar, Sreekanth J. Varma, Jayan Thomas, Swaminathan Rajaraman

**Affiliations:** 1 Department of Materials Science & Engineering, University of Central Florida, 4000 Central Florida Blvd, Orlando, FL 32816, USA; chart@knights.ucf.edu (C.H.); kowsik@knights.ucf.edu (K.K.); Jayan.Thomas@ucf.edu (J.T.); 2 NanoScience Technology Center, University of Central Florida, 12424 Research Parkway, Suite 400, Orlando, FL 32826, USA; avra.kundu@ucf.edu (A.K.); dr.sreekanthvarma@gmail.com (S.J.V.); 3 Department of Physics, Sanatana Dharma College, Alappuzha, Kerala 688003, India; 4 CREOL—College of Optics and Photonics, University of Central Florida, 4000 Central Florida Blvd, Orlando, FL 32816, USA; 5 Department of Electrical & Computer Engineering, University of Central Florida, 4000 Central Florida Blvd, Orlando, FL 32816, USA; 6 Bridging the Innovation Development Gap (BRIDG), 200 Neocity Way, Kissimmee, FL 34744, USA

**Keywords:** nanostructured Interdigitated Electrodes, shadow mask, cardiomyocytes, in vitro cellular analysis

## Abstract

Adverse cardiac events are a major cause of late-stage drug development withdrawals. Improved in vitro systems for predicting cardiotoxicity are of great interest to prevent these events and to reduce the expenses involved in the introduction of cardiac drugs into the marketplace. Interdigitated electrodes (IDEs) affixed with a culture well provide a simple, suitable solution for in vitro analysis of cells because of their high sensitivity, ease of fabrication, and label-free, nondestructive analysis. Culturing human pluripotent stem cell differentiated cardiomyocytes onto these IDEs allows for the use of the IDE–cell combination in predictive toxicity assays. IDEs with smaller interdigitated distances allow for greater sensitivity, but typically require cleanroom fabrication. In this communication, we report the definition of a simple IDE geometry on a printed nanostructured substrate, demonstrate a Cellular Index (*CI*) increase from 0 to 7.7 for human cardiomyocytes, and a decrease in *CI* from 2.3 to 1 with increased concentration of the model drug, norepinephrine. The nanostructuring results in an increased sensitivity of our 1 mm pitch IDEs when compared to traditionally fabricated IDEs with a pitch of 10 μm (100 times larger electrode gap). The entire nanostructured IDE (nIDE) is fabricated and assembled in a rapid nanofabrication environment, thus allowing for iterative design changes and robust fabrication of devices.

## 1. Introduction

Drug-induced cardiotoxicity accounts for one-third of safety-based withdrawn pharmaceuticals, making it the number one cause of drug withdrawal, limitation, and development termination [1,2,3]. As of 2016, the Tufts Center for the Study of Drug Development estimates the cost of developing a new drug is on average 2.89 billion US dollars [4]. Because of this high cost, improved in vitro systems for predicting drug-induced toxicity are of great demand in the pharmaceutical industry to decrease late-stage drug attrition, advance rapid development, and reduce monetary loss [2,3].

Interdigitated electrodes (IDEs), which are comprised of two individually addressable, interwoven, comb-like electrode structures, are one of the most favorable and widely used transducers as chemical and biological sensors because of their low cost, high sensitivity, and ease of fabrication [5]. By affixing a culture well to the IDE substrate, a biosensor can be easily fabricated. This allows for cells to be cultured onto the surface and assessed with label-free electrical and optical assays. A low-voltage signal induces a current between the IDEs. The cells on the electrodes at the bottom of the culture well impede this current, and a change in impedance results [1]. Measuring this impedance change across these electrodes gives an indirect measure of the number of cells in each culture well, as well as an assessment of the interaction between the cells and electrodes [2]. Cellular impedance measurements are useful for studying cell growth and drug interactions in vitro without the use of destructive labelling procedures with fluorescent, chemiluminescent, or radioactive chemicals [6]. Recently, these efforts are gaining industrial acceptance with efforts of collaboration between various electrode manufacturers to introduce rapid assays with uniform standards across industrial and academic testing laboratories for testing cardiotoxicity [7].

Such predictive toxicity assays based on human pluripotent stem cells may aid in predicting potential safety issues of drug candidates early in their development process, provide information about the mechanisms of drug-induced organ toxicity, reduce the reliance on animal testing, and increase the relevance of preclinical safety tests [2,3,7]. Human induced pluripotent stem cell (iPSC) differentiated cardiomyocytes are the ideal candidate for cardiotoxicity cell-based studies. They exhibit the molecular and functional properties of an intact human heart, and their electrical signatures can be monitored using nondestructive impedance sampling [2].

Several IDE and impedance-based biosensors exist, but many of them require the use of expensive commercial systems [1,2,3,8,9,10,11,12,13] for data analysis and involve cost-prohibitive, cleanroom-based fabrication approaches for the IDE micro- and nanostructuring [14]. These systems use very densely packed electrodes, which cover a majority of the substrate surface and prohibit optical tracking of cells. Other approaches integrate microelectrode arrays with IDEs, which allows for more comprehensive measurement at the cost of more complex fabrication processes [15].

Typical methods for the fabrication of nanostructures include methods like photolithography [16], e-beam lithography [17], and focused ion beam lithography [18]. Even though these methods offer high-quality nanostructures, they involve tedious procedures, long processing time, limited scalability, and high cost. To achieve scalability, bottom-up approaches like self-assembly have been used, but they are limited to select materials, and pattern versatility cannot be easily achieved using this approach. Sacrificial anodic aluminum oxide (AAO) templates for developing nanostructures are also widely used [19] for fabricating nanostructures; however, the sacrificial nature of AAO and the required use of strong chemical etchants place a serious limitation on this method. Several unconventional lithographic methods have been developed to circumvent the limitations posed by conventional lithographic techniques. Among these techniques, nanoimprinting lithography (NIL) [20] has attracted considerable attention. In NIL technique, many nanostructures can be replicated using an inexpensive NIL machine from a master mold. The feature size depends on the mold used to print the nanostructures. These nanostructures can subsequently be used as substrates for various applications, including interdigitated electrodes.

Here we present a new impedance-based sensor that allows for long-term in vitro cellular analysis with high fidelity. IDEs are placed atop a nanostructured polyacrylonitrile (PAN) substrate, whose geometry is designed to maximize the interaction with the electrodes and cells. As a result, the device is fashioned as nanostructured Interdigitated Electrodes (nIDEs). Both the IDE and the nanostructured PAN substrate are fabricated utilizing “Rapid Micro/Nanofabrication Approaches.” This results in cost effectiveness, rapid translation from design to a fabricated device, utilization of direct write techniques, and the ability to reduce drug candidate testing times by an order of magnitude or more with dramatically increased sensitivity despite coarser geometries. The hypothesis being evaluated in this rapid communication article is that the interaction of the nanostructured PAN substrate with the electrodes should increase the sensitivity of the IDEs, and as a result, electrodes with a larger pitch should have the same performance as electrodes that are orders of magnitude smaller. In addition, we demonstrate the utility of the nIDE for cardiotoxicity screening by testing it with varying concentrations of a model drug.

## 2. Materials and Methods

The section below describes the design and fabrication of the nIDE device, followed by the details of the assays and impedance measurements.

### 2.1. Design of the nIDEs

**Figure 1:**
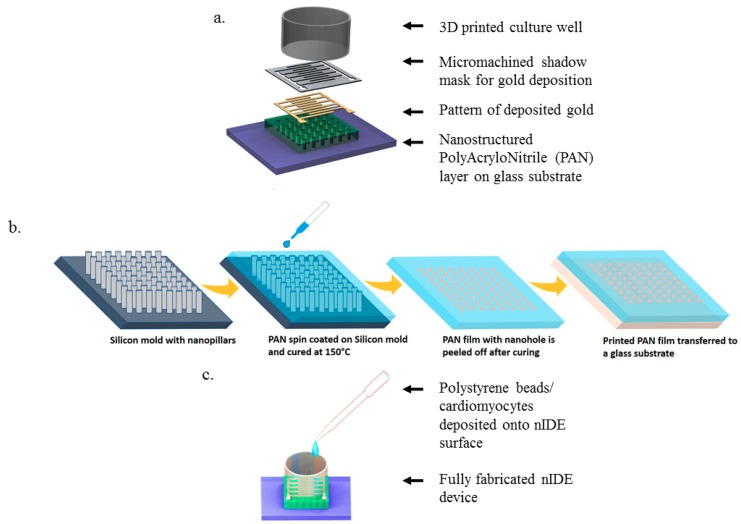
nIDE Nanofabrication and Device Assembly: (**a**) Schematic of nIDE device: A nanostructured polyacrylonitrile (PAN) layer is affixed to a glass substrate. A gold Interdigitated Electrode (IDE) structure is then patterned on top using a shadow mask. Finally, a 3D-printed culture well coated with PDMS is attached to enable the IDE to be used with biological specimens; (**b**) Process flow for spin-on nanoimprinting (SNAP) fabrication of PAN nanostructures; (**c**) Fully assembled device being plated with polystyrene beads/cardiomyocytes.

SolidWorks (Dassault Systems, Waltham, MA, USA) was used to design the interdigitated electrodes and the culture wells. A schematic of the nIDE device and its components are depicted in Figure 1a–c. The gold IDEs are designed to be 800 μm wide and 1 mm long with a pitch of 1 mm and a thickness of 30 nm. The nanostructured chip is comprised of a 5 mm by 5 mm nanomachined PAN layer of 10 μm thickness with 50 nm nanoholes with a pitch of 200 nm. The fully assembled chip is designed to be 10 mm by 10 mm.

### 2.2. Fabrication of the PAN Nanostructures

Recently, we developed a spin-on nanoimprinting process (SNAP), shown in Figure 1b, to make nanostructures in a very short time [21,22,23]. A polymer solution is prepared by dissolving 8 wt.% of polyacrylonitrile (PAN, Mw = 150,000) in dimethylformamide and heated at 150 °C for 5 min. The PAN solution was then cooled and spin-coated on a premade Si mold with the inverse of the pattern to be printed. The PAN film was subsequently peeled off from the mold, transferred to a glass substrate, and cured at 250 °C. SNAP technique does not need high temperature, pressure, or any other force during the printing process. We have successfully developed nanoholes as small as 50 nm on PAN films to make IDEs. The silicon mold fabrication is a one-time event and several hundreds of nanostructures can be printed using the SNAP process from the same master mold without any degradation [23].

### 2.3. Fabrication of the nIDEs

The 1 mm pitch IDE shadow masks were fabricated using the IDE designs by micromilling. A 90-degree T-8 Mill Tool (150–250 µm diameter; T-Tech, Peachtree Corners, GA, USA) was spun at 55,000 rpm in a T-Tech J5 Quick Circuit Prototyping System to micromill through an 80 μm thick stainless-steel sheet (Trinity Brand Industries, Countryside, IL, USA).

The shadow masks were affixed to the 5 mm by 5 mm squares of nanomachined PAN atop a glass carrier substrate using Kapton**^®^** tape (Dupont, Wilmington, DE, USA). A metal film comprised of 30 nm of gold was deposited onto the nanostructured PAN/glass substrate (Figure 1a) using electron beam evaporation (Thermionics Laboratory Inc., Hayward, CA, USA) to metallize the interdigitated electrodes. The shadow mask was released carefully after the metallization step to reduce the damage to the underlying nanostructures. To package the nIDE device, a 3D-printed (Form 2, Formlabs, Somerville, MA, USA) culture well (10 mm inner diameter, 10 mm tall) was dip coated with polydimethylsiloxane (PDMS) in its entirety to improve biocompatibility of the printed resin material [24], and attached to the substrate using biocompatible 353ND epoxy (EpoTek, Billerica, MA, USA) [25]. Lead wires of sanded copper were subsequently attached to the contact pads using an E2101 electrically conductive silver epoxy (Epotek, Billerica, MA, USA).

### 2.4. Polystyrene Bead Assay

Prior to the cardiomyocyte assay, polystyrene (PS) latex beads of 1.1 μm particle size and concentration of 0.1 mg/mL in deionized (DI) water (Sigma Aldrich, St. Louis, MO, USA) were used to emulate a cell-like material (Figure 1c). The aqueous suspension of PS latex beads was diluted with DI water in a ratio of 1:10 and the diluted solution was uploaded into a syringe and transferred to the culture well of the IDE device. The impedance measurements, using a frequency sweep from 10 Hz to 10 MHz, were performed on an IDE fabricated on a PAN nanomachined layer with a nanohole diameter of 50 nm and a pitch of 200 nm using a BODE Impedance Analyzer (Omicron Labs, Klaus, Austria). The reported impedance values are an average of two devices (N = 2).

### 2.5. Cell Culture

Human induced pluripotent stem cell (iPSC) differentiated cardiomyocytes (iCell Cardiomyocytes^2^, Cellular Dynamics, Madison, WI, USA) were used for cell studies. The iPSC cardiomyocytes were kept frozen in liquid nitrogen until they were cultured according to the manufacturer’s directions [26]. To ensure that the cells adhered to the surface, the IDE devices were coated with 5 μL of 1:20 fibronectin (Sigma Aldrich, St. Louis, MO, USA) and Dulbecco’s Phosphate-Buffered Saline (DPBS) without Calcium and Magnesium (Gibco, Waltham, MA, USA) solution and placed in an incubator (37 °C with 7% CO_2_) for one hour. Cells were thawed and counted to determine the density. This task was performed by mixing 100 μL of cells suspended in media with 0.4% Trypan Blue (Fisher Scientific, Waltham, MA, USA). A droplet of 100 μL of this solution was subsequently applied to a glass hemocytometer and placed under a 10 times microscope objective of a Nikon TE200 Inverted Fluorescence Microscope (Nikon, Tokyo, Japan) for observations. Live, unstained cells were counted in each of four sets of sixteen squares. The cell counts from each of the four sets of squares were averaged and multiplied by 10,000 and then multiplied by 5 to correct for the 1:5 dilution from the Trypan Blue addition. The fibronectin was aspirated, and the thawed cells were plated onto the nIDE surfaces (N = 8) and two six-well plates (control Polystyrene Plates from Corning Inc., Corning, NY, USA) and incubated for one hour (at 37 °C with 7% CO_2_). A measured droplet of 300 μL of iCell Cardiomyocytes maintenance medium (Cellular Dynamics, Madison, WI, USA) was subsequently added to each of the eight culture wells and the controls. Full media changes occurred every other day.

Eight nIDE devices were densely plated with iCell cardiomyocytes with an average cell density of 310,500 cells per culture well. Each of the two six-well plates (controls, N = 12 wells) were plated with an average cell density of 50,000 cells per well.

### 2.6. Biocompatibility Assay

After one day in vitro (DIV01), nanostructured PAN was placed in the six-well plate that was plated with approximately 50,000 cardiomyocytes. Biocompatibility studies were performed in the six-well plates at DIV07 (days in vitro) to ensure that the nanostructured PAN surface was suitably compatible with the iCell cardiomyocyte cell line. This study was performed using the Promega Cell-Titer Glo Luminescent Cell Viability Assay Kit (Promega, Waltham, MA, USA). A volume of reagent measuring 0.5 mL was added to each culture well and mixed for two minutes to induce lysis. The six-well plate was incubated at room temperature for ten minutes to stabilize the subsequent measurement of a luminescent signal. Luminescence was recorded using a Tecan Infinite Pro 200 plate reader (Tecan, Männedorf, Switzerland) with the emission wavelength set at 500 nm, the excitation wavelength set at 365 nm, and an integration time of 10 s. The background and control measurements with only media in the culture well and media with cells in the culture well, respectively, were performed in addition to the nanopatterned PAN measurements.

### 2.7. Impedance Measurements

Electric cell-substrate impedance sensing (ECIS) was used to characterize the electrochemical properties of the cell-substrate interface. A low-voltage signal is applied to the nIDEs, which forms ionic currents in the cell culture medium. When cells are located on the nIDEs, these ionic currents are affected by the number, morphology, and adhesion of these cells [10]. Impedance will gradually increase during the normal cell growth and proliferation process; thus, as more cells become attached to the nIDEs, an increase in electrical impedance is expected.

For the impedance measurement, a full spectrum of the frequency range from 10 Hz to 10 MHz, with an AC voltage perturbation of 20 mV, was scanned using BODE 100 impedance measurement station (Omicron Labs, Klaus, Austria) from DIV07 to DIV18. The measurements started at DIV07 since we optically observed coverage of the nIDE with the cardiomyocytes. Impedance was normalized using the cell index (*CI*) calculation [12,13], which is represented in Equation (1), where Δ*Z* is the change in real part of impedance from day to day and *Z_o_* is the background impedance on the nIDE.

(1) CI=ΔZZo

Since drug-induced cardiotoxicity is of great interest with these biosensors, impedance measurements were additionally performed on the nIDEs with cultured iPSC cardiomyocytes utilizing different concentrations of norepinephrine (Sigma Aldrich, St. Louis, MO, USA) as a model drug compound. Concentrations of norepinephrine ranging from 0 μM to 250 μM were introduced to the culture well at DIV 21 and impedance was measured to detect any changes due to the addition of the model drug. Cell index results from the model drug experiments were further normalized by using the percent cell index (%*CI*) [12,13], represented in Equation (2), where *CI_o_* is the cell index with no norepinephrine and *CI_c_* is the cell index for a specific concentration of norepinephrine.

(2) %CI=CIcCIo

Normalization of cell index calculations is an established calculation for such assays with IDE-based approaches and this task was performed to compare our approach with others in this space.

## 3. Results and Discussion

**Figure 2:**
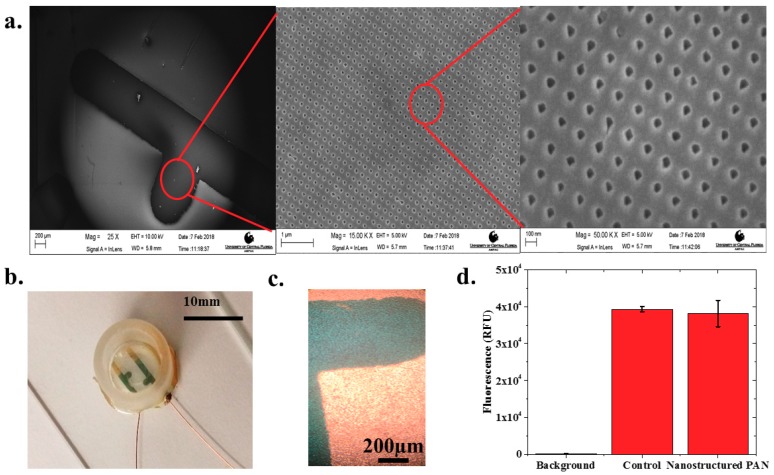
(**a**) SEM image of electrodes deposited on top of the nanostructured PAN layer with zoomed-in SEM images of the printed 50 nm PAN nanohole structures. Scales from left to right are 250 μm, 1 μm, and 180 nm, respectively; (**b**) Completed nIDE device with culture well and wires attached; (**c**) Human iPSC Cardiomyocytes cultured on 1 mm pitch nIDE at DIV01. The cardiomyocytes completely cover the surface of the IDE; (**d**) Biocompatibility assay results: the nanostructured PAN substrate shows similar (97.01% ± 2.15%) biocompatibility (N = 6) to control samples comprised of just cells in a six-well plate.

Nanostructured IDEs (nIDEs) were successfully fabricated on the nanostructured PAN substrates. The nanostructures on the PAN substrate remained defined after the deposition of the gold IDE structure, as shown in Figure 2a. These SEM micrographs clearly depict the nanostructured PAN with repeatable “nanoholes” of approximately 50 nm in diameter and the mm-scale gold electrodes defined on top of these “nanoholes”. The gold IDE structures measured within 99.37% of the designed dimensions representing an excellent translation from design. Fully assembled devices, as shown in Figure 2b, remained intact throughout the entire life cycle of the cardiomyocytes (DIV18).

Human iPSC cardiomyocytes were successfully cultured onto the nIDEs as depicted in a sample image in Figure 2c. One can clearly observe a mat of cells on top of the IDEs in the images collected with transmitted light microscopy. The cell viability assay confirmed that the nanostructured PAN substrate was cytocompatible for cell culture with iCell Cardiomyocytes ^2^. The other components of the nIDE (gold, PDMS, and 353ND epoxy), have previously been established to be cytocompatible with cardiomyocytes [24,25]. Fluorescence levels were well above the background fluorescence, which indicates that most of the cultured cells were viable, as shown in Figure 2d. Quantitatively, the nanostructured PAN and control wells (N = 6 for both types) both showed a fluorescence of nearly 40,000 RFU with a low standard deviation of approximately 3000 RFU, which depicts excellent cytocompatibility performance (97.01% ± 2.15% of the control) with iCell Cardiomyocytes^2^.

**Figure 3:**
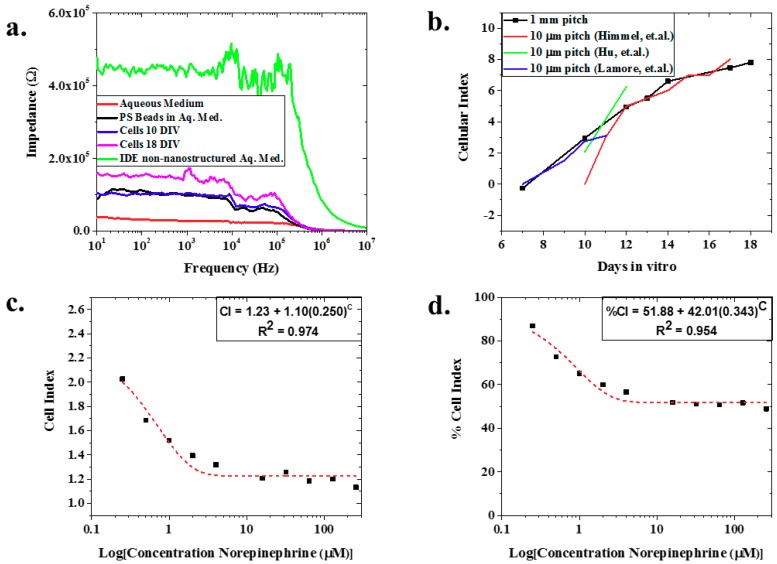
(**a**) Representative full spectrum impedance measurements—the nIDEs with cardiomyocytes cultured on them show an increased impedance from the nIDEs that only have an aqueous medium. In addition, as the cells proliferate, an increase in impedance is observed; (**b**) Variation of the Cellular Index (CI) of the cells cultured on the nIDEs for 18 days: it was observed that the *CI* of the nIDEs increased over time as expected because the cell coverage of the nIDE increased over time. The calculated mean value of N = 8 wells of nIDEs is represented by the square bullet point. A comparison to the data from Himmel et al. tracks the *CI* changes observed in our assay. Since our IDE pitch is 100 times the pitch demonstrated by the IDE from Himmel, we believe the presence of the nanostructures results in improvement in device sensitivity; (**c**) *CI* and (**d**) %*CI* for norepinephrine experiment: Mean values for N = 7 is depicted with a square bullet point. The *CI* and %*CI* both show an exponential decrease, with excellent fits given by their R^2^ values of 0.974 and 0.954, respectively, as the concentration of norepinephrine increased because the cells were dying due to the dosage of the drug. This caused a departure from the impedance of the nIDE with no norepinephrine added.

Figure 3a depicts an example of the raw, full spectrum impedance data for polystyrene beads, iCell Cardiomyocytes^2^ (average value of N = 8 at two specific days: DIV10 and DIV18), and aqueous media clearly delineating the three analytes. Further, this figure shows that the impedance of the nIDEs with cells (110.19 kΩ at 1 kHz for DIV10 and 243.21 kΩ at 1kHz for DIV18) and polystyrene beads (96.53 kΩ at 1 kHz) was higher than the nIDE with just aqueous medium (27.37 kΩ at 1 kHz), which follows expected trends due to the modification of the ionic currents due to the presence of cells and the PS beads [1]. In addition, Figure 3a demonstrates a decrease in impedance from an IDE without a nanostructured substrate to that of the nanostructured IDE, both in aqueous media. Looking closely at the ECIS for the two sample DIV measurements (N = 8), they demonstrated an increase in impedance as the days in vitro increased. The cell index also demonstrated an increase as the days in vitro increased (Figure 3b). This is an expected result as reported by Himmel et al. The morphological changes in the cells lead to an increased impedance as the cell coverage increases, and the cell coverage will inherently increase as the cells grow over time [1,27]. This result held until the assay was terminated at DIV18. To our knowledge, we are the first to report this result with 1 mm pitch nIDEs over a period of 18 days with human cardiomyocytes, and a very similar trend using a commercial system with 10 μm pitch IDEs on a glass substrate fabricated with a complex photolithographic technique involving several steps [27] is reported by Himmel, Hu et al., and Lamore et al. [1,3,28]. Our devices are 100 X larger than these three other studies but report similar *CI* increases from cell growth (*CI* = 1.1 on DIV10 increasing to *CI* equal to approximately 7–8 on DIV17). We believe the comparable performance is due to the nanoscale structure patterned onto the substrate of the nIDEs. As a result, we believe that the nIDE shows an increased sensitivity compared to commercial IDE systems, which have an electrode gap that is 100 times smaller. We believe that this increase in sensitivity is due to the increased electrode surface area provided by the nanoholes. When surface area increases, capacitance increases; thus, impedance decreases, giving greater sensitivity [29,30,31], as shown in Equation (3), where C is the capacitance, *ε_o_* is the vacuum permittivity, *ε_r_* is the relative permittivity of the electrolyte, *A* is surface area of the electrode, *d* is electrode gap, and *Z* is impedance.

(3) $$C=\frac{\varepsilon_{o}\varepsilon_{r}A}{d}\propto \frac{1}{Z}$$

The model drug experiment with norepinephrine showed a decrease in both cell index (from *CI* = 2.34 at 0 μM of norepinephrine to *CI* = 1.13 at 256 μM norepinephrine) (Figure 3c) and percent cell index (86.91% at 0 μM of norepinephrine to 48.88% at 256 μm norepinephrine) (Figure 3d) with increasing concentrations of norepinephrine (mean of N = 7). Both the *CI* and %*CI* for norepinephrine addition demonstrate exponential behavior with excellent fits of 0.974 and 0.954, respectively. This is expected because as the cardiomyocytes are exposed to higher concentrations of norepinephrine, more cells are expected to die from the exposure [32]. As a result, there was less active cellular coverage, which lowers the impedance, *CI*, and %*CI*.

## 4. Conclusions

This study reports for the first time to our knowledge the development of IDEs patterned onto nanostructured PAN substrates using rapid micro/nanofabrication technologies. The resulting nanostructured IDEs (or nIDEs) demonstrated excellent design to device (99.37% translation of design features) and biocompatibility of 97.01% ± 2.15% with respect to iCell cardiomyocytes^2^ control. The nIDEs were developed as a tool for rapid screening of toxins with an impedance metric and they demonstrated an impedance (110.19 kΩ at 1 kHz for DIV10 and 243.21 kΩ at 1 kHz for DIV18) that was higher than the IDEs with just an aqueous medium (27.37 kΩ at 1 kHz) which was used as a control. In addition, the nIDEs with cells showed increased impedance as evidenced by a Cell Index (CI) increase from 0 to 7.7 with increasing days in vitro of cell culturing. This result is as predicted because impedance should increase as cell coverage increases because of the cell–electrode interaction. Long-term cell culture (DIV18) was demonstrated with iCell Cardiomyocytes^2^ and, most significantly, an improvement in device performance when fabricated on nanostructured substrates (100 times larger than commercial systems) was demonstrated with cellular index calculations. Finally, the cardiotoxicity testing utility of our devices was successfully demonstrated with the expected response of decreased cellular index from 2.34 to 1.13 in response to increased concentrations of a model drug, norepinephrine.
